# Coseismic deformation analysis of the 2017 Milin Ms 6.9 earthquake in the Namche Barwa Syntaxis: Implications for regional tectonics

**DOI:** 10.1016/j.fmre.2024.09.003

**Published:** 2024-09-17

**Authors:** Junyi Wang, Shishu Zhang, Youjia Zhao, Fulong Cai, Chao Wang, Jiankun He, Lin Ding

**Affiliations:** a State Key Laboratory of Tibetan Plateau Earth System, Environment and Resources, Institute of Tibetan Plateau Research, Chinese Academy of Sciences, Beijing 100101, China; b University of Chinese Academy of Sciences, Beijing 100049, China; c POWERCHINA Chengdu Engineering Corporation Limited, Chengdu 610072, China

**Keywords:** Namche Barwa syntaxis, Xixingla fault, Joint inversion, Numerical simulation, Coulomb stress

## Abstract

The 2017 Milin earthquake occurred near Namche Barwa on the southeastern Tibetan Plateau. This research employed Sentinel-1 imagery to capture the corresponding coseismic deformation field and adopted a joint inversion approach using geodetic measurements and teleseismic waveform data to assess earthquake mechanics. The coseismic deformation indicated distinct differential movements, indicating a thrust movement of this fault with uplift in northeast side. The calculated seismic moment magnitude was Mw 6.5, with the fault striking of 305° and a dip angle of 72° The earthquake hypocenter was located at a depth of approximately 10 km, and the maximum fault slip was approximately 1.08 m. The event was attributed to the northwestern segment of the Xixingla fault. Numerical simulations indicated a peak ground velocity of approximately 0.3 m/s. The northwestern segment of the Xixingla fault was identified as the seismogenic fault of the event. Furthermore, the Coulomb stress analysis indicates that the earthquake induced stress loading on the Dongju–Milin and Jiali faults. The surface strain analysis reveals a region of high strain adjacent to the fault. The 2017 Milin earthquake occurred because the Xixingla fault underwent predominantly thrust-type sliding due to the continuous tectonic stress induced within the Tibetan Plateau. Notably, the middle segment of the Xixingla fault currently experiences relatively low seismic activity and is in a state of stress and strain accumulation. Thus, strong earthquakes may occur within the middle segment of the Xixingla fault.

## Graphical abstract

**Figure:**
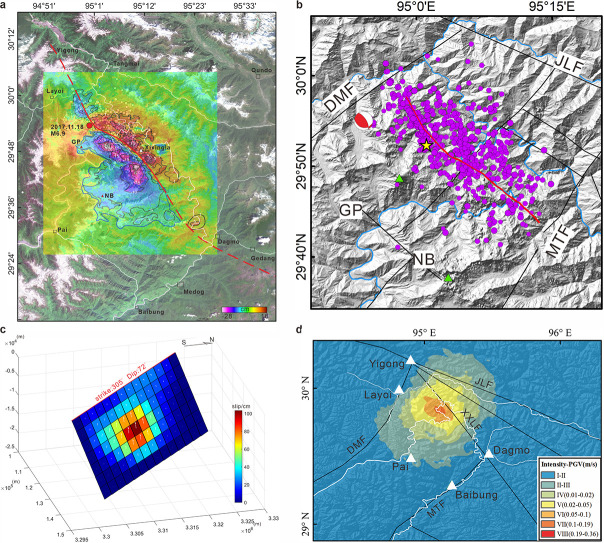

## 1 Introduction

The collision between the Indian and Eurasian plates led to the formation of the Himalayan orogenic belt [1,2]. Located at the southeastern end of the Himalayan orogenic belt, the eastern tectonic junction is divided into the northern Lhasa block and the southern Namche Barwa syntaxis by the Yarlung Tsangpo suture belt [3]. The eastern tectonic junction stands out as one of the most active zones of tectonic deformation in the Tibetan Plateau and is distinguished by notable local compression and uplift of crustal material. The crust in this area is more than 60 km thick, and due to the subduction of the Indian plate, rocks undergo high-grade metamorphism and high-temperature ductile deformation [4,5]. Several types of faults surround the tectonic junction, including the Jiali and Xixingla faults, which strike southeast, as well as the Dongju-Milin and Motuo-Aniqiao faults, which strike northeast [3,5]. In addition, this region encompasses the two highest peaks in southeastern Tibet—Mount Gyala Peri (GP, 7294 m) and Mount Namche Barwa (NB, 7782 m)—between which the Yarlung Tsangpo River flows, carving out the renowned Yarlung Tsangpo Grand Canyon with an elevation decrease of more than 2 km.

The Namche Barwa syntaxis is regarded as a thrust structure beneath the Yarlung Zangbo suture belt [6]. West of the Namche Barwa syntaxis lies the Milin strike-slip fault, east lies the Aniqiao–Motuo strike-slip fault, and north lies the Jiali strike-slip fault [3]. The Jiali right-lateral strike-slip fault, situated in the southeastern Tibetan Plateau, serves as the southern boundary for the southeastward lateral extrusion of the plateau and plays a crucial role in shaping the structural deformation of the region [7]. The Jiali fault can be divided into three distinct segments on the basis of its spatial distribution and activity levels [8]. These segments include the northwest segment extending from Naqu to Jiali, the middle segment spanning Jiali to Yigong and Tongmai, and the southeast segment extending from Bomi to Chayu [9]. Furthermore, the southeastern segment is further subdivided into the Palung Zangbo fault, the Gongrigabuqu fault, and the Xixingla fault [9]. Paleoseismic records show that the middle and southeastern segments of the Jiali fault have experienced at least five paleoseismic events since the late Quaternary, with earthquake recurrence intervals ranging from 2000 to 5000 years [8,10]. The Xixingla fault, also known as the Xixingla-Dagmo fault [9] or the Bianba-Dagmo fault [11], extends southeastward from the Yigong earthquake swarm to the area near the epicenter of the 1950 M8.6 Chayu earthquake. GPS data have shown that the Xixingla fault has slip rates of 8–10 mm/yr north of the Namche Barwa syntaxis [11] and 10–15 mm/yr south of Dagmo.

**Fig. 1:**
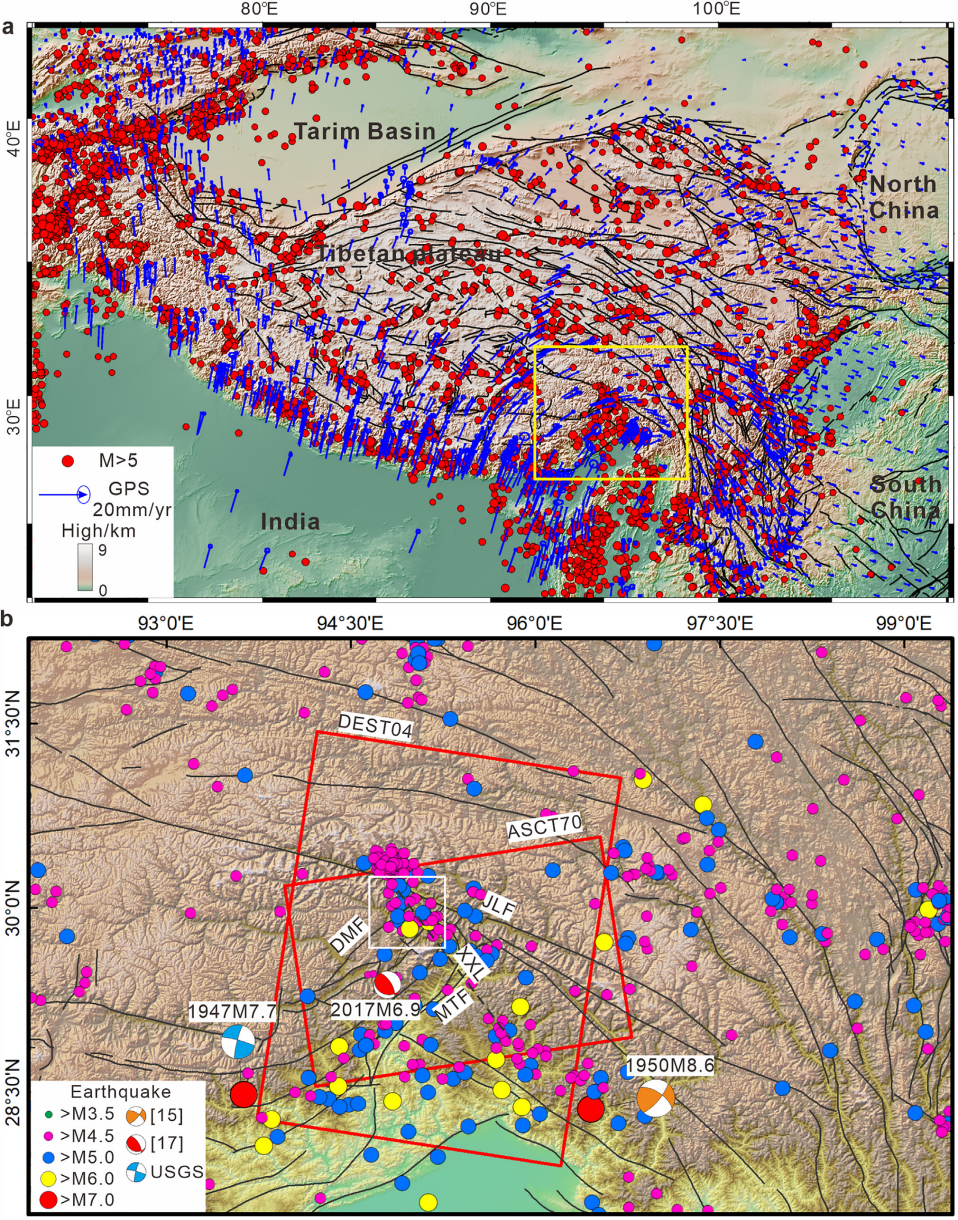
(a) GPS horizontal velocity field of the Tibetan Plateau; GPS data from [12,13]. Seismic activity of the Tibetan Plateau from 1900 to 2023 (earthquake catalog from the USGS, https://earthquake.usgs.gov/). (b) Seismic activity in the eastern structural region from 1900 to 2023. The red line outlines the Sentinel-1 image track. The source mechanism for the 1950 Chayu earthquake is from Li et al. [15], and that for the 2017 Milin earthquake is from Bai et al. [17]. JLF: Jiali fault, DMF: Dongjiu–Milin fault, XXLF: Xixingla fault, MTF: Motuo fault.

The global positioning system (GPS) horizontal velocity field (Fig. 1a) suggests a clockwise rotation of deformation within the Tibetan Plateau, particularly around its eastern tectonic junction [12,13]. The western boundary of the Eastern Himalayan region primarily exhibits left–lateral strike‒slip motion [14], with the northern boundary showing primarily thrust motion with a minor strike‒slip component. This contrasts with the eastern boundary's predominant right-lateral strike-slip motion. With respect to the vertical velocity field, the Namche Barwa syntaxis has experienced rapid exhumation since the late Miocene [3]. The complex tectonic setting and significant crustal deformation in the region have led to frequent earthquakes [[15], [16]]. Since 1900, multiple earthquakes with magnitudes greater than Mw 6.0 have occurred in the area (Fig. 1b).

On November 18, 2017, a significant Ms 6.9 earthquake struck near the Namche Barwa syntaxis in Milin, Tibet [17]. According to preliminary studies, the earthquake was triggered by the Xixingla fault [18]. The Xixingla fault is adjacent to the large glaciers between Mount Gyala Peri and the intermontane valleys [5,17]. In the context of this event, several organizations, including the United States Geological Survey (USGS, https://earthquake.usgs.gov/), the Global Centroid Moment Tensor (GCMT, https://www.globalcmt.org), and the China Earthquake Networks Center (CENC, http://www.ceic.ac.cn), provided focal mechanisms for the earthquake (Table 1), agreeing that the 2017 Milin earthquake was a thrust event. However, significant differences are present in the earthquake characterizations among these institutions, with variations in reported magnitude and focal depth also noted.

**Table 1:** **Focal mechanisms from different studies**.

Source	Magnitude	Strike/°	Dip/°	Rake/°	Dpeth/km	Maxslip/m
USGS	Mw 6.4	303	36	83	11.5	—
GCMT	Mw 6.5	328	66	108	12	—
CENC	Ms 6.9	310	63	90	10	—
[19]	Mw 6.4	—	—	—	9	1.9
[20]	Mw 6.5	—	—	—	10.5	0.83
[21]	Mw 6.45	308	76	82.2	9	0.51
This study	Mw 6.5	305	72	88	10	1.08

Researchers initially identified the seismogenic fault of the 2017 Milin earthquake via interferometric synthetic aperture radar (InSAR), GPS, aftershock, and seismic waveform data [[18], [19], [20], [21], [22], [23], [24], [25]]. However, significant differences are present in the geometric parameters and slip distributions of the faults. These discrepancies can be attributed to the limited constraints from geodetic measurements or seismic data and to the variations in inversion methods and strategies employed in different studies (Table 1). First, there are discrepancies in the interpreted dip angles of the fault surface. Some scholars, using InSAR data to invert the geometric parameters of faults, have suggested a southwestward inclination for the mainshock [19,20]. Conversely, other researchers have examined the characteristics of aftershock distributions and inferred a northeastward fault inclination on the basis of InSAR data [18,21,25]. Moreover, Xiong et al. [25] proposed a bivergent fault rupture model for this earthquake. Second, there is considerable variation in the reported slip distributions along the fault plane. Different inversion methods have yielded results ranging from 0.3 to 1.9 m in size on the basis of InSAR inversion [19,21,25], joint InSAR and GPS analysis [18], and combined InSAR and teleseismic waveform data inversion [20]. Furthermore, while some studies have focused primarily on seismic activity along the Xixingla fault, research on other aspects of the fault remains relatively scarce [23,24]. Research reveals that in regions characterized by dramatic topographical changes, numerical simulations of strong ground motions are likely to be affected [26]. The pronounced variations in terrain within the Namche Barwa syntaxis region notably influence the numerical simulations associated with earthquakes.

This study uses Sentinel-1 images and aftershock data to ascertain the fault deformation characteristics of the 2017 Milin earthquake. By jointly inverting InSAR, GPS, and teleseismic P-wave data, the fault parameters and coseismic slip of the seismogenic fault can be determined. By developing a model that integrates the influence of terrain factors and employing numerical simulations of strong ground motions, surface acceleration and peak ground velocity during earthquakes can be obtained, enabling the analysis of surface motion processes and seismic risk associated with seismic events. This paper begins with an introduction to the coseismic deformation and fault distribution characteristics of the 2017 Milin earthquake, followed by a detailed description of the joint inversion of multiple datasets to determine the fault characteristics of the earthquake. Finally, on the basis of the regional Coulomb stress and surface strain rate results, the deformation features and tectonic mechanisms of the earthquake were examined, and the Xixingla fault was further analyzed. This study provides in-depth insights into fault movement related to the 2017 Milin earthquake, thereby advancing the understanding of the seismic activity and tectonic background of the eastern tectonic junction.

## 2 Materials and methods

### 2.1 Coseismic deformation

To acquire the coseismic deformation associated with the 2017 Milin earthquake, we processed Sentinel-1 ascending and descending images that covered the event area (Table S1). We performed terrain phase removal and geocoding using 30-m-resolution data from the Shuttle Radar Topography Mission (SRTM). Phase unwrapping was performed with the minimum cost flow algorithm. The vertical component of the coseismic deformation in the LOS direction was determined [27] by calculating the incidence angle between the satellite line of sight (LOS) and the Earth's surface [28]. Consequently, on the basis of the incidence angles of the satellites during the ascending and descending passes of the 2017 Milin earthquake, the vertical deformations of different orbital deformation fields were computed separately. To calculate the coseismic three-dimensional deformation field, we implemented a strain model (SM) with variance component estimation (VCE) for weighting to increase the estimation accuracy of the three-dimensional surface deformation [[27], [28], [29]].

### 2.2 Joint inversion

InSAR and GPS data can not only provide information on near-field surface deformation but also effectively constrain the spatial distribution of subsurface faults. Seismic waveform data can generally be categorized into near-field and teleseismic waveform data. Teleseismic waveform data contain low-frequency components that record the characteristics of seismic waves propagating over long distances [30]. Consequently, teleseismic waveform data are beneficial for in-depth investigations of the Earth's internal structure and propagation patterns of seismic waves [20]. Furthermore, owing to their smaller incidence angles, teleseismic waveform data are conducive to accurate and reliable inversion of seismic source depths [30]. The utilization of teleseismic waveform data has been widely applied in the inversion of earthquake fault slip [20,30]. Fig. 1 reveals that the coseismic deformation pattern displayed by the ascending InSAR data is more regular than that depicted by the descending data. Consequently, in the inversion process, we exclusively utilized the ascending data. The InSAR data underwent uniform downsampling prior to inversion, resulting in a total of 3081 data points. For the integration of teleseismic observations, we selected 28 broadband P-wave records from globally distributed stations that exhibited high signal‒to‒noise ratios and an approximately uniform azimuthal distribution within the epicentral distance range of 30°–90° Additionally, bandpass filtering ranging from 0.02 to 0.1 Hz was applied to the waveform data to mitigate long-period noise potentially caused by complex geological structures and short-period signal interference. For the GPS data, we selected 6 near-field stations that recorded coseismic horizontal displacements [18].

### 2.3 Numerical simulation

The numerical simulation of strong ground motion is crucial for understanding the areas of maximum intensity around the epicenter and predicting the characteristics of future significant earthquakes [31]. Near-field ground motion is a primary cause of seismic disasters, making the calculation of earthquake-induced acceleration vital for seismic mitigation [32]. The peak ground velocity, which directly influences the deformation and stress conditions of buildings, is commonly used to assess its impact on structures, infrastructure, and land [33]. To obtain accurate ground acceleration and peak ground velocity results, we conducted numerical simulations of strong ground motion via CDEM software on the basis of slip data obtained through joint inversion. Research has revealed a significant amplification effect of topography on the propagation of seismic waves [26]. Given the rugged terrain of the Namche Barwa region, we established a model incorporating terrain undulations to simulate the strong ground motion of the 2017 Milin earthquake more accurately.

## 3 Results

### 3.1 Coseismic deformation of the 2017 Milin earthquake

**Fig. 2:**
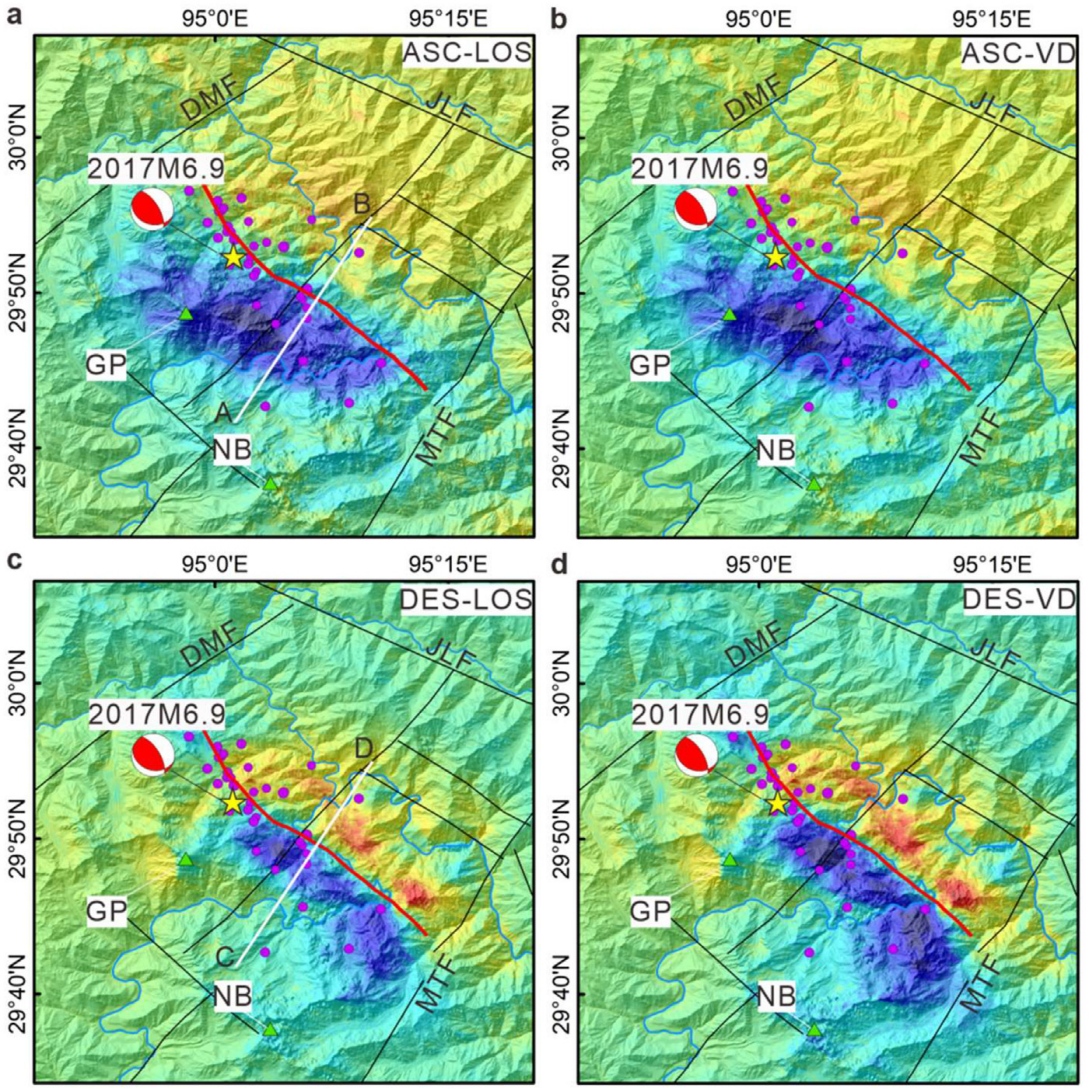
(a, b) Ascending LOS and vertical deformation. (c, d) Descending LOS and vertical deformation. The red dots indicate the distribution of aftershocks within one hour of the 2017 Milin earthquake, data from Peng et al. [22].

On the basis of the coseismic deformation results obtained from InSAR (Fig. 2a-d), the seismogenic fault clearly prominently extends in a northwestern direction. In both the ascending and descending InSAR deformation fields, the deformation values in the southwestern block are consistently negative. However, in the descending deformation results (Fig. 2c, d), some regions within the northeastern block of the fault show positive deformation values. These observations agree with the InSAR findings of Liu et al. [21], who reported uplift on the northeastern side and subsidence on the southwestern side of the fault. To further analyze the characteristics of the deformation field, we extracted deformation data along transect lines that intersect the ascending and descending InSAR deformation fields (Fig. 2a, c). These profiles consistently show subsidence on the southwestern side of the fault (Fig. S1) and uplift on the northeastern side. These coseismic deformation patterns suggest that the northeastern block underwent uplift, whereas the southwestern block experienced subsidence, indicating the typical characteristics of deformation due to an earthquake along a thrust fault.

**Fig. 3:**
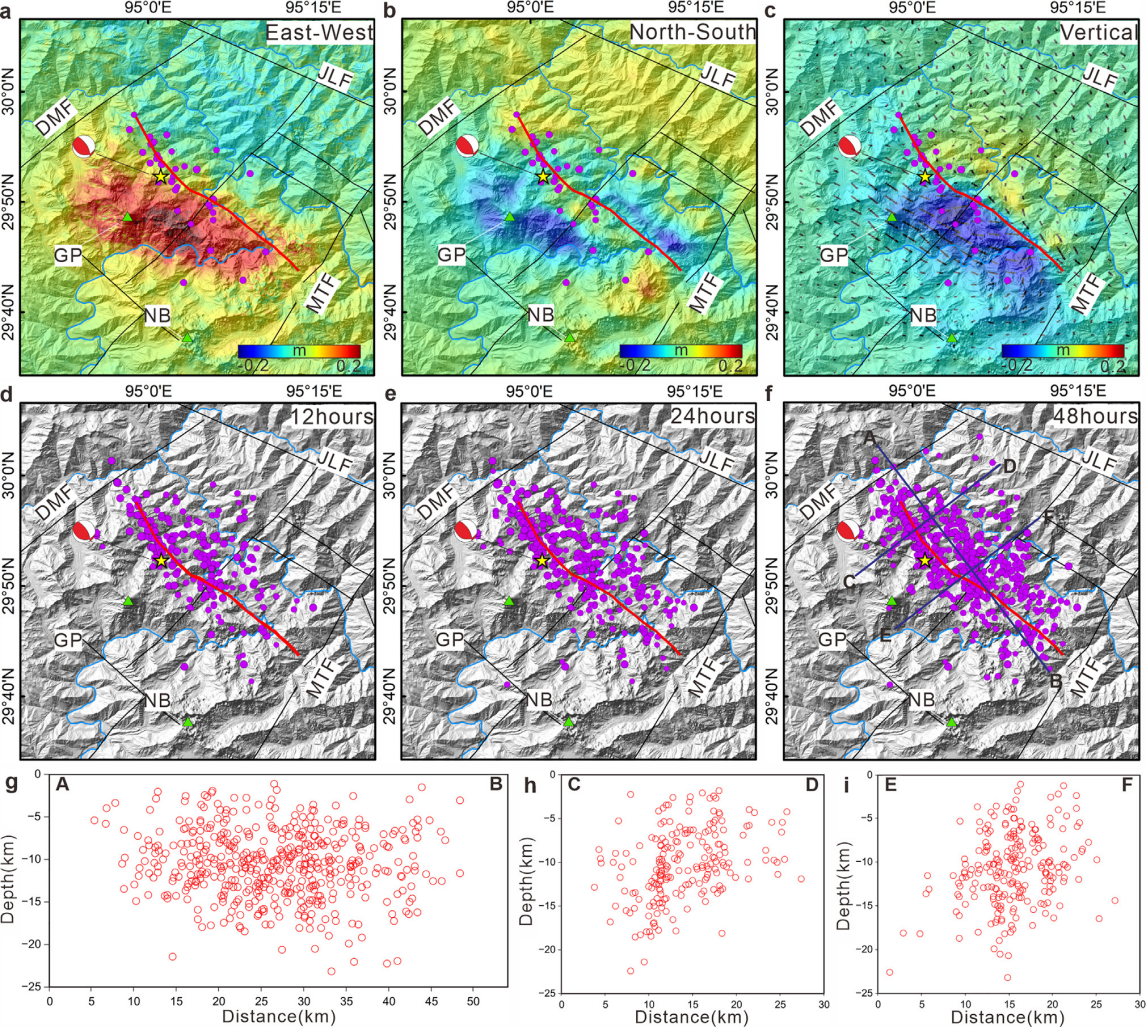
(a) East‒west deformation. (b) North‒south deformation. (c) Vertical deformation. (d-f) Aftershock occurrence within 12 h, 24 h and 48 h following the mainshock. (g-h) Seismic distributions along profiles AB, CD and EF.

Using the SM–VCE method, we calculated the three-dimensional surface deformation based on the LOS and vertical deformation data from the ascending and descending orbits for the 2017 Milin earthquake (Fig. 3a-c). The results indicate that the surface deformation from the 2017 Milin earthquake was primarily concentrated in the east‒west and vertical directions. The vertical deformation reveals significant subsidence in the southwestern block of the fault. The GPS coseismic deformation field of the event indicates that horizontal displacement was directed toward the epicenter [18], exhibiting the characteristics of seismic movement along a thrust fault. The three-dimensional surface deformation results indicate that an earthquake can be characterized as a thrust event. Notably, the subsidence deformation in the southwestern block of the fault exceeds the uplift deformation in the northeastern block.

To determine whether the seismogenic fault of the 2017 Milin earthquake actually comprises two distinct faults, we analyzed the distribution of aftershocks across various periods to reveal the temporal evolution of the earthquake sequence. The results of the analysis indicate that the aftershocks were predominantly distributed in the northwesterly direction nearly parallel to the Xixingla fault ( Fig. 3). These aftershocks were localized to a narrow strip, measuring approximately 42 km in length and 26 km in width, centered on the main shock. Notably, within the first hour following the mainshock (Fig. 3a-c), the majority of aftershocks were recorded along a single fault segment on the southwestern wall of the coseismic deformation zone (Fig. 3). Three profile lines were subsequently drawn across fault, and the aftershocks were projected onto these profiles. The section analysis demonstrated (Fig. 3g-i) that the majority of the aftershocks were localized at depths ranging from 3 to 15 km. The aftershock distribution results from Wang et al. [23] also suggest that aftershock sequences occurred along high-dip-angle fault. Research by Jian et al. [18] revealed that the mainshock of the 2017 Milin earthquake originated from a single fault, which initiated subsequent fault ruptures.

### 3.2 Source parameters and fault slip distribution

On the basis of the characteristics of the surface deformation field and the aftershock distribution, we can infer that the fault strike of the 2017 Milin earthquake was oriented northwestward (Fig. 3). Additionally, considering the moment tensor solutions from the global centroid moment tensor (GCMT) catalog and the actual geological structures, we establish the focal plane of an initial model with strike, dip, and rake angles of 310°, 66°, and 83°, respectively. We treated the surface as the upper boundary of the fault rupture and constructed an initial model of a finite fault with a length of 42 km and a width of 21 km, dividing the fault plane into subfaults, each measuring 3 km by 3 km. For the calculation of Green's functions, we utilized the crust1.0 model [34] to establish a layered viscoelastic model and applied the normalized calculation method proposed by Wang [35] to derive the Green's functions for seismic wave records. To integrate seismic waveform data and static geodetic measurements, we employed a joint inversion approach for the finite fault rupture process, which is based on both seismic waveform and static geodetic data [30]. At the start of the joint inversion, we set the maximum rupture velocity to 3 km/s for the subfaults. Furthermore, to determine the relative weights of the different datasets, we empirically set the weight ratio of the P-wave, GPS, and InSAR data to 1:0.5:0.5 according to a series of calculations.

**Fig. 4:**
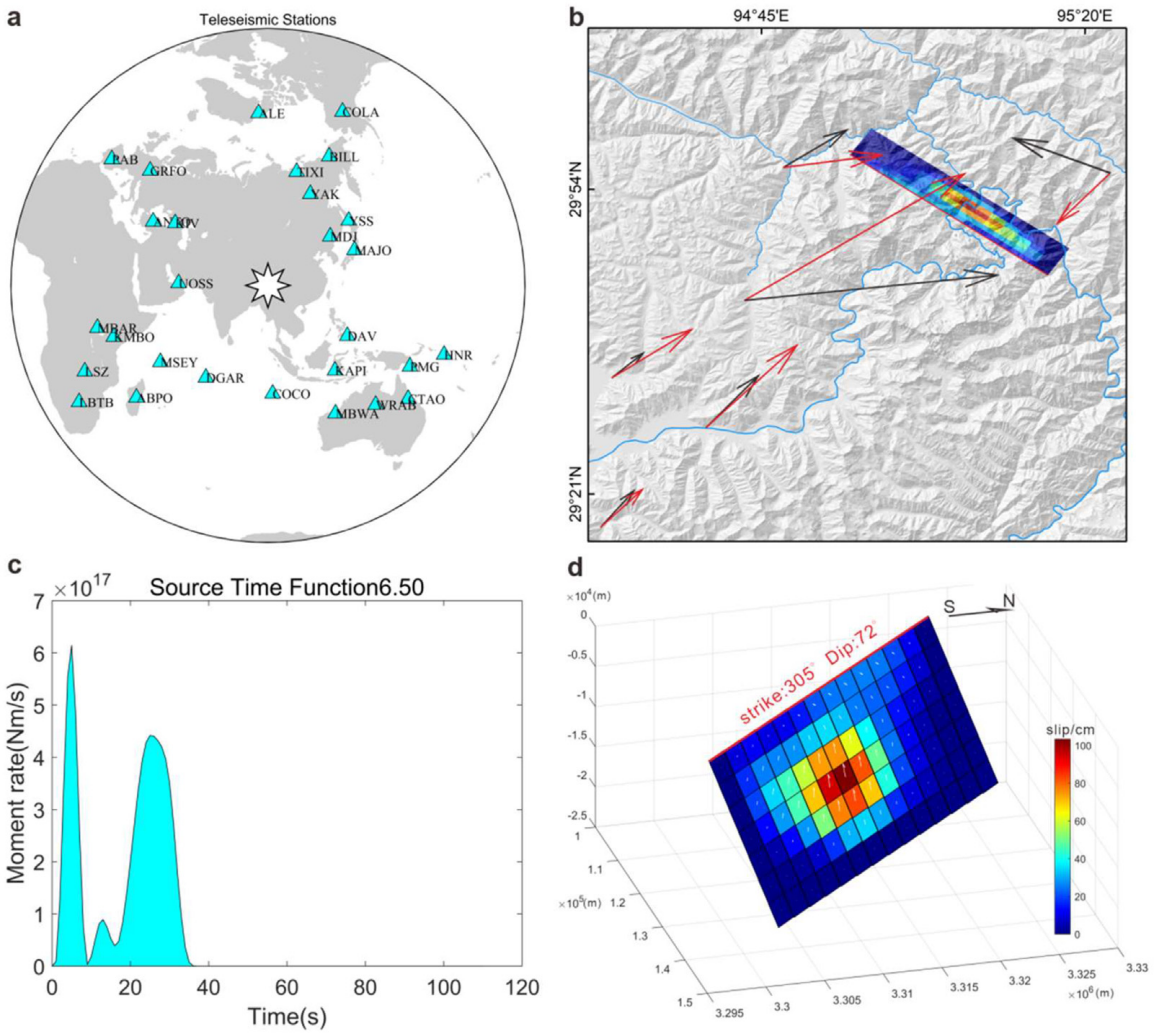
**The joint inversion results of the InSAR, GPS, and teleseismic P-waveform data are presented**. (a) The 2017 Milin earthquake and teleseismic stations (data from IRIS, http://ds.iris.edu/wilber3/find_event). (b) Comparison of observed (black) and simulated (red) GPS data and GPS data from [18]. (c) Fault source–time function. (d) Distribution of fault slip.

The final results of the joint inversion indicate that the fault slip associated with the 2017 Milin earthquake was characterized primarily by thrust motion (Fig. 4). The inversion results of the InSAR and waveform datasets are consistent with the observational findings (Fig. S2), suggesting that the developed fault model corresponds to the actual fault. The inversion analysis reveals a fault strike of 305°, a dip angle of 72°, an average rake angle of 88°, and a moment magnitude of Mw 6.5. The maximum slip displacement along the fault reached approximately 1.08 m, occurring at a depth of approximately 10 km. The most significant slip along the fault was localized within a depth range of 5 to 12 km (Fig. 4d). Steeply dipping thrust kinematics of the Milin earthquake, seismic deformation was primarily concentrated in the vertical direction. However, the GPS coseismic deformation data gathered in this study encompass only horizontal components. Moreover, given the complexity of the terrain in the research area and the limitations associated with simplified single-fault models, disparities between the observed and simulated values exist on the northeastern side of the fault. These results suggest a shallow seismic source for the earthquake, with the majority of ruptures occurring in the upper crust. The observations were limited to phenomena such as sand liquefaction, surface cracks, and rock collapses during the field survey (Fig. S3), and no other apparent surface rupture features were detected. The fault may not have fully ruptured to the surface.

### 3.3 Numerical simulation of strong ground motion

**Fig. 5:**
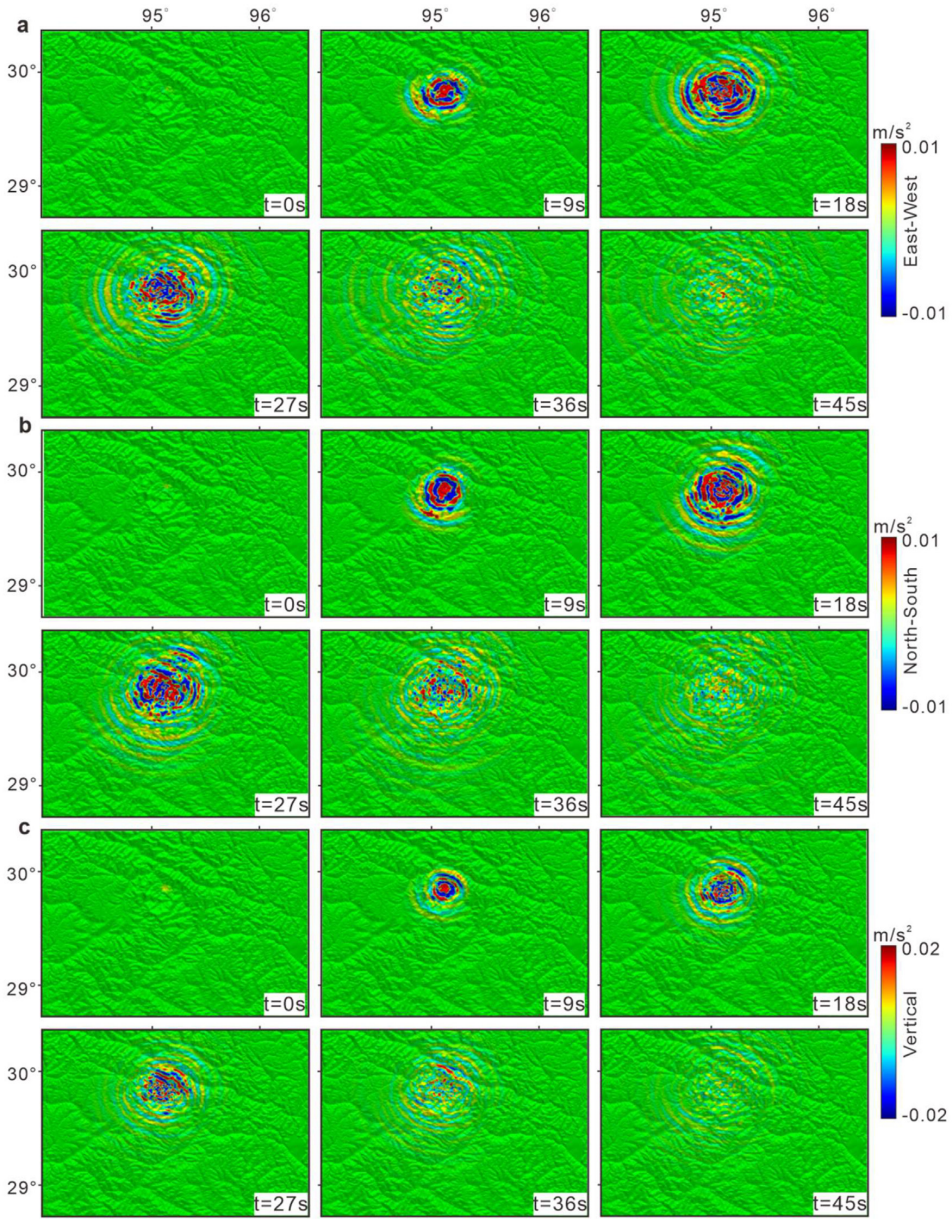
**Surface acceleration**. (a) East‒west acceleration. (b) North‒south acceleration. The east‒west and north‒south accelerations represent the characteristics of the horizontal ground motion of seismic waves. (c) Vertical acceleration. The vertical acceleration signifies the vertical movement of seismic waves at the surface.

The surface acceleration results (Fig. 5) demonstrate significant peak variations across different locations. The east‒west and north‒south accelerations represent the characteristics of the horizontal ground motion of seismic waves, whereas the vertical acceleration signifies the vertical movement of seismic waves at the Earth's surface. This classification not only aids in accurately describing the trajectory of seismic waves but also provides significant insights for research on fault activity. The surface accelerations in the east‒west direction (Fig. 5a) and north‒south direction (Fig. 5b) indicate pronounced horizontal movements, primarily propagating toward Mount Gyala Peri and Mount Namche Barwa on the southwestern side of the fault. Conversely, the vertical direction results (Fig. 5c) reveal a smaller spatial extent of seismic wave propagation than the horizontal direction, with the waves primarily concentrated between the epicenter and the area around Mount Gyala Peri and Mount Namche Barwa.

In Fig. 5, the three component fields exhibit similar patterns. This phenomenon stems from the fact that thrust fault earthquakes often involve large-scale stress release. Such stress is typically generated in relatively uniform compressive environments, consequently leading to a more consistent distribution of stress waves in all directions. Therefore, this uniform stress distribution results in acceleration fields displaying similar patterns across different directions. The surface acceleration analysis reveals that the intricate and varied terrain in the study area significantly amplifies seismic waves, which primarily propagate toward the southwestern edge of the fault. This result is consistent with the deformation field distribution obtained through InSAR, indicating greater deformation along the southwestern edge as well.

**Fig. 6:**
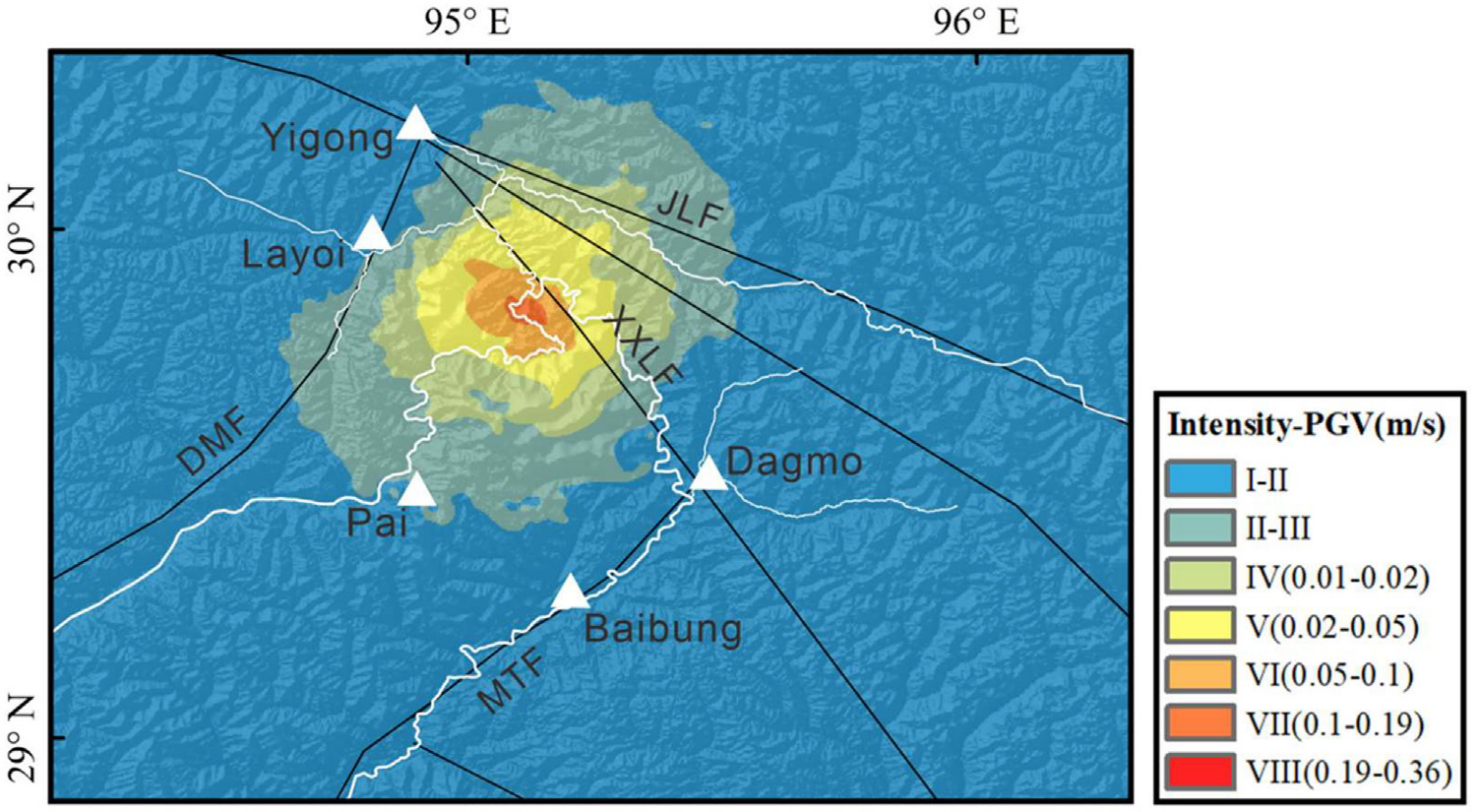
**Peak ground velocity (PGV) of the Milin earthquake**.

The peak ground velocity results (Fig. 6) indicate that the maximum peak ground velocity in the epicentral area exceeds 0.3 m/s. Areas experiencing an intensity greater than VI are located primarily in the sparsely populated region where the Yarlung Tsangpo Grand Canyon. He et al. [36] conducted a study on the postseismic slope deformation of the Milin earthquake via PSI-InSAR technology. These findings reveal a significant increase postseismic slope deformation rates within the epicentral area. This suggests that the seismic event not only caused surface destruction during the quake but also continued to affect geological stability in the seismic zone. Postseismic activity may lead to mountain instability, landslides, and other secondary disasters. By integrating the results of peak ground velocity and postseismic deformation monitoring, it is evident that the Milin earthquake significantly impacted the Mount Gyala Peri region.

### 3.4 Coulomb stress

**Fig. 7:**
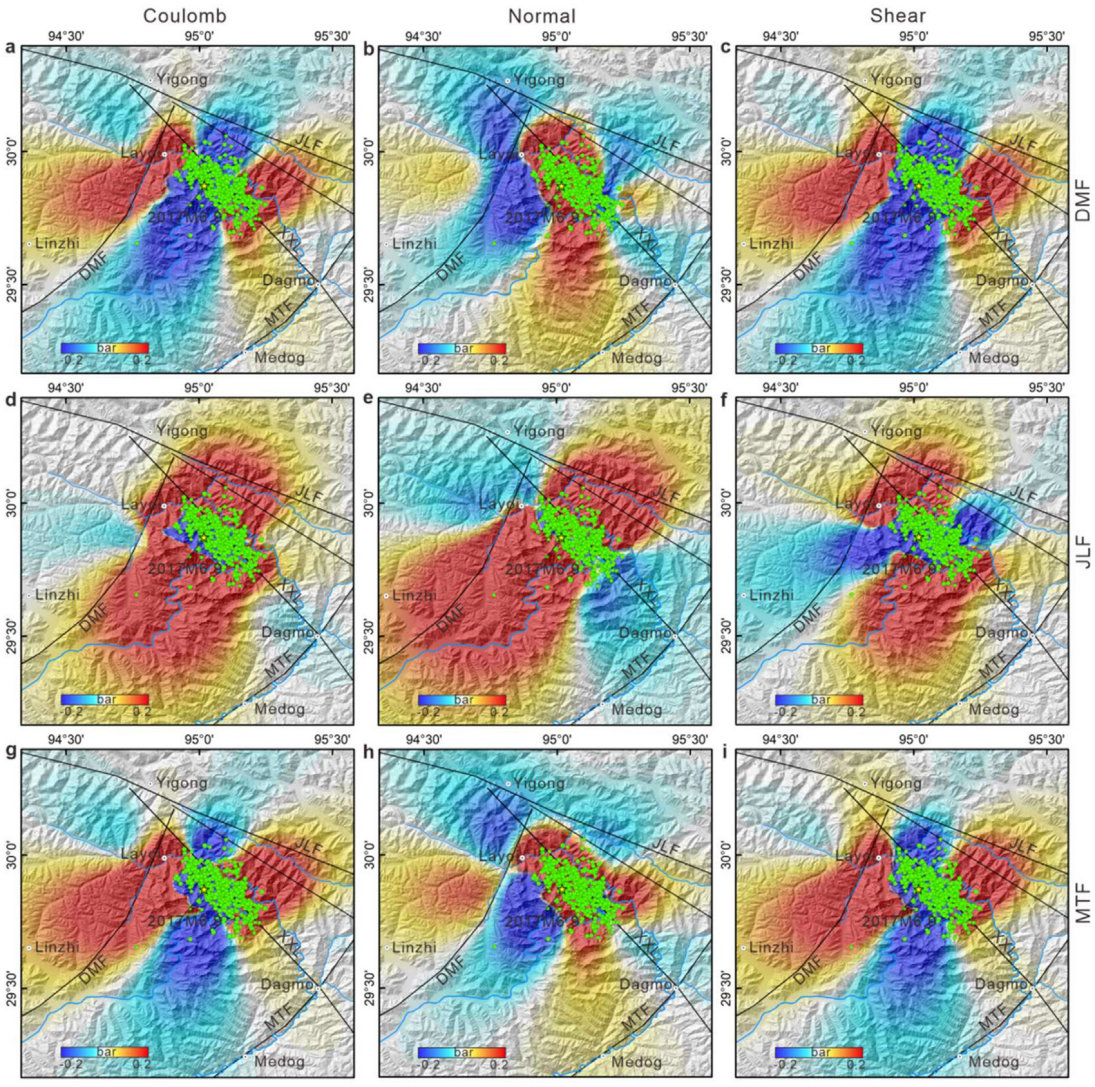
**The various receiver faults associated with coseismic Coulomb stress, normal stress, and shear stress are illustrated for different faults**. (a-c) Stress distributions with the Dongjiu–Milin fault acting as the receiver fault. In these panels, the variations in the Coulomb stress, normal stress, and shear stress are analyzed to understand the seismic response of the fault. (d-f) Corresponding stress distributions for the Jiali fault as the receiver fault, demonstrating how the stress changes in this fault system under seismic activity. (g-i) Effects on the Motuo fault, providing a comparative view of the stress alterations in response to seismic events. These figures collectively offer insights into the stress transfer mechanisms and their implications for seismic hazard assessment in the respective fault regions.

During an earthquake, rupture on a fault can alter the stress distribution within the region, potentially triggering or delaying seismic events on neighboring faults [37]. Yin et al. [38] determined the Coulomb stress imparted by the 1950 Chayu earthquake and reported that it impacted the stress on the seismogenic fault of the 2017 Milin earthquake, potentially precipitating its earlier occurrence. To assess the impact of the 2017 Milin earthquake on regional stress changes, we used the Coulomb 3.3 software package to compute the stress variations induced on surrounding faults by this earthquake [39,40]. By selecting source fault parameters from the inversion results of the 2017 Milin earthquake, we identified the Dongjiu–Milin fault, the Jiali fault, and the Motuo fault as receiver faults and computed the changes in Coulomb stress, normal stress, and shear stress on those faults. Considering that the maximum slip occurred at a depth of 10 km, we assessed stress changes at this depth for the various faults, adopting a friction coefficient of 0.4 on the basis of relevant empirical studies [40]. The 2017 Milin earthquake had a discernible impact on several faults near the epicenter. Our findings suggest that the Coulomb stress changes attributable to the 2017 Milin earthquake led to stress loading effects on the Dongjiu-Milin and Jiali faults (Fig. 7), whereas its impact on the Motuo fault was less significant. In scenarios where we treated the Jiali fault as the receiver fault, the earthquake markedly increased both the normal and shear stresses on the Xixingla fault. The northeastern segment of the Xixingla fault experienced an increase in Coulomb stress, whereas the effect on the more distant Motuo fault was comparatively limited.

## 4 Discussion

### 4.1 Deformational differences between the hanging wall and footwall of the fault

In this section, we first discuss the rupture features observed on the ground and then discuss the InSAR observations to provide a comprehensive interpretation of the deformational differences. During the field survey, observations were limited to phenomena such as sand liquefaction, surface cracks, and rock collapses; no other apparent surface rupture features were detected. Owing to the upper plate effect associated with thrust-type earthquakes, the seismic motion of the hanging wall of a fault tends to be greater than that of the footwall [21]. However, the InSAR deformation field data from the 2017 Milin earthquake reveal the opposite phenomenon. The InSAR data demonstrate that the northeastern hanging wall underwent less uplift than the observed subsidence on the southwestern footwall. Hu and Yao [41] utilized ambient noise tomography to determine regional crustal velocity structures and revealed the presence of a high-velocity area to the north, west, and east of the 2017 Milin earthquake, suggesting relatively rigid crust. Additionally, Li et al. [20] discovered that the southwestern foot wall of the 2017 Milin earthquake lies within a low-density region, primarily located at depths of 5–10 km within the crust. The block in the southwestern part of the seismic zone consists mainly of hornblende rocks [18]. Moreover, Han et al. [42] calculated the b value in the region, revealing higher b values for the southeastern block of the fault, which indicates relatively brittle rock characteristics. After a comprehensive analysis, it was concluded that the influence of regional crustal lithology caused the 2017 Milin earthquake to result in more substantial fracturing of crustal material in the southwestern block, which is situated within the crust's brittle zone. As a result, more deformation occurred in the southwestern block than in the northeastern block.

### 4.2 Characteristics of the Xixingla fault distribution

Although previous researchers have conducted preliminary studies on the fault of the 2017 Milin earthquake, there are still certain shortcomings [[18], [19], [20], [21], [22], [23], [24], [25]]. The diverse fault solutions in these studies can be attributed to several factors. One primary reason is the different data sources and inversion methodologies employed. For example, InSAR data provide high-resolution spatial information but may experience decorrelation in vegetated or developed areas, whereas GPS data offer precise point measurements but lack spatial coverage. Combining these methods, while beneficial, can still introduce variance on the basis of weighting and error handling in the inversion process. The inversion techniques themselves also play a crucial role. Some studies may prioritize data fitting over physical plausibility, or vice versa, which can lead to different fault models. Studies using joint inversion approaches seek a balance between multiple data types, often resulting in more robust models, but these models are also more complex and sensitive to the initial assumptions and weighting schemes used. Our work advances interpretation by leveraging more comprehensive datasets and refined inversion techniques, enhancing the reliability of fault models compared with earlier methods.

The coseismic deformation field derived from InSAR analysis indicates that the seismogenic fault of the 2017 Milin earthquake was the northwestern segment of the Xixingla fault. Zeitler et al. [43] analyzed 16 months of seismic network data from the western boundary of the Namche Barwa syntaxis and the Jiali fault and identified 1254 seismic events. These events were mainly situated above a depth of 20 km within the upper crust and distributed along a steeply dipping fault, together forming a distinct linear seismic belt along the southern edge of the Jiali fault. Bai et al. [17] argued that the seismogenic fault of the 2017 Milin earthquake corresponds to the Xixingla fault, with evidence of activity since the Holocene. Wang et al. [23] located two fault structures in the aftershock sequence of the 2017 Milin earthquake, which dip northeastward and are nearly parallel to the Xixingla fault. Furthermore, research by Hua et al. [44] via ambient noise tomography revealed that the faulting mechanism of the 2017 Milin earthquake involved a series of imbricate thrust faults. Notably, Xie et al. [24] proposed that an active northwestern fault zone extends from the Namche Barwa syntaxis to the Chayu block.

**Fig. 8:**
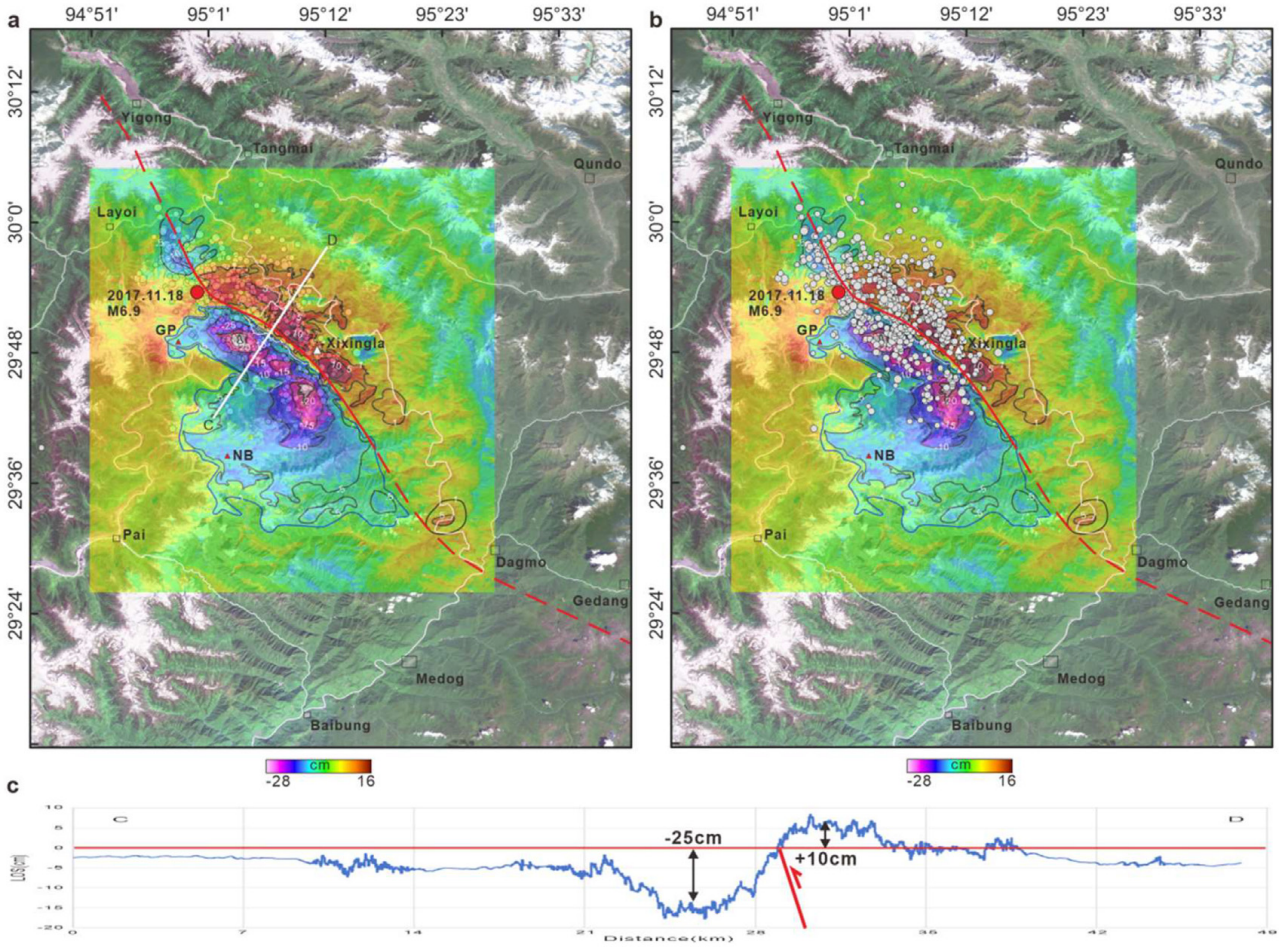
**Spatial distribution characteristics of the 2017 Milin earthquake seismogenic fault**. Based on the results of descending InSAR deformation and the distribution of aftershocks, the spatial distribution of the Xixingla fault is identified. The white dots represent the aftershocks of the Milin earthquake. (a) The descending InSAR deformation result layer is positioned above, while the aftershocks layer is positioned beneath it. (b) The aftershocks layer is positioned above, with the descending InSAR deformation result layer beneath it. (c) The line-of-sight deformation characteristics are identified based on the descending InSAR deformation results and fault inversion results.

However, field surveys are limited by the complexity of geological conditions and extensive vegetative cover. To analyze the spatial characteristics of potential seismogenic faults, seismic events from 1900 to 2023 were compiled from sources such as the USGS, the CENC, and Xie et al. [24]. Earthquake events were then mapped onto high-resolution satellite images to study the distribution of the Xixingla fault (Fig. S4). These events revealed that the Xixingla fault stretches continuously from Namche Barwa to near the site of the 1950 Chayu earthquake, forming a pronounced seismic zone, as depicted in Fig. S4. This zone comprises three distinct seismic clusters located at Yigong, on the northern flank of Mount Namche Barwa, and on the southeastern segment of the fault. The central portion, particularly near Dagmo, shows a paucity of seismic events. Further insights were gained by superimposing the descending coseismic deformation field and aftershocks onto the satellite imagery (Fig. 8); the resulting contours closely correspond to the aftershock distribution. The northeastern margins of both Mount Namche Barwa and Mount Gyala Peri follow the trace of the Xixingla fault. From Layoi, the fault extends southeastward, delineating an 'S' shape as it crosses the Yarlung Tsangpo River, eventually converging near the 1950 Chayu earthquake zone. This analysis revealed that the Xixingla fault, which experienced frequent seismic activity along its course from Yigong to near Chayu, was the source fault of the 2017 Milin earthquake.

### 4.3 Mechanism of high-angle thrust earthquakes

The comparison of fault geometric parameters between our study and others reveals (Table 1) that the 2017 Milin earthquake has a dip angle ranging from 63° to 76°, which is significantly greater than that of most thrust earthquakes. However, in regions experiencing intense tectonic activity, a conducive tectonic environment can facilitate the occurrence of high-angle thrust earthquakes [25]. For example, the 2001 Bhuj Mw 7.5 earthquake in western India, as determined through InSAR data inversion, exhibited a dip angle of 51° [45]. The seismic inversion results of the 2016 Nura Mw 6.5 earthquake revealed that it occurred on a steep north-dipping fault with a dip angle of 67° [46]. Furthermore, research on the 2018 Hokkaido Eastern Iburi Mw 6.6 earthquake indicated a remarkably high dip angle of 74°, classifying it as a high-angle thrust earthquake [47].

The occurrence of the 2017 Milin earthquake suggested that this event was a consequence of the continuous collision between the Indian Plate and the Eurasian Plate, resulting in predominantly thrust motion on the Xixingla fault under the tectonic stress induced by the eastern Himalayas. The focal mechanism reveals that this earthquake was a high-angle thrust event, reflecting the predominance of compressive strain in the structural characteristics of the region. These findings are consistent with intense crustal shortening and uplift processes along the southeastern margin of the Tibetan Plateau. The earthquake resulted in significant differential movements on either side of the Xixingla fault, with uplift on the northeast side and subsidence on the southwest side, exhibiting typical features of a thrust fault. Notably, the subsidence on the southwest side surpasses the uplift on the northeast side, potentially attributable to the relative brittleness of the crustal rocks in the southwest region. Comprehensive analysis suggests that the earthquake was a product of structural deformation along the southeastern margin of the Tibetan Plateau, with postseismic effects potentially accelerating strain accumulation on other active faults in the region, thereby increasing regional seismic risk.

The Xixingla fault, situated at the apex of the great bend of the Yarlung Tsangpo River, intersects with the Yarlung Tsangpo Suture Zone to the south and terminates at the strike-slip Jiali fault to the north. To the west, it is adjacent to Mount Gyala Peri and the extensive glaciers developing in its intermontane valleys, demarcating the southern boundary of the southeastward extrusion of the Tibetan Plateau. Geological data suggest that in the vicinity of the Namche Barwa syntaxis, the Dongjiu-Milin fault and the Xixingla fault collectively regulate the strike-slip component of the syntaxis [[1], [21]]. The focal mechanism of the 2017 Milin earthquake aligns well with the Xixingla fault, revealing the structural activity characteristics dominated by large-scale compression and dextral lateral extrusion in the eastern part of the orogenic belt.

### 4.4 Regional surface strain rate

Owing to the intense collision and compression between the Indian plate and the Eurasian plate, the eastern tectonic junction has undergone extensive deformation [3]. The Namche Barwa syntaxis has been subjected to intense tectonic compression and rapid uplift. The Xixingla fault is located in a region characterized by significant compressive and shearing forces. To elucidate the strain characteristics of the eastern tectonic junction, we incorporated GPS data [12,13] to compute the regional surface strain rate (Fig. 1a). The surface strain field can be quantified via GPS data, which offers insights into the surface strain features related to seismic activity [48]. By using the interpolation method with elastic constraints for two-dimensional vector data [48], we used Strain_2D software to calculate dilation rates and maximum shear stress [49]. With respect to the dilatational field results (Fig. S5a), the positive and negative color bands indicate volume expansion and contraction, respectively, in the corresponding regions. Fig. S5a shows that the expansion rate decreases continuously from the Namche Barwa syntaxis southeastward to the epicenter of Xiachayu. This shows that the eastern Himalayan region is subjected to a state of high strain. For the maximum shear stress (Fig. S5b), the data reveal that the shear stress peaks near the epicenter of the 1950 Chayu earthquake. From the findings regarding both the expansion rate and the maximum shear stress, one can infer that the Xixingla fault lies within a region characterized by intense compression and shear deformation.

## 5 Conclusion

In this study, we processed InSAR coseismic deformation data to calculate the three-dimensional surface deformation field. Constrained by InSAR and GPS geodetic measurements, we used aftershock data to determine the characteristics of the seismogenic fault and inverted the source parameters and slip distributions via teleseismic waveform data. The results suggest that the 2017 Milin earthquake occurred as a thrust event along a high-dip-angle fault segment striking in the northwestern direction. The hanging wall of this fault is on the northeast side, and the footwall on the southwest side experiences more subsidence deformation than the corresponding uplift deformation of the hanging wall. The Xixingla fault was identified as the seismogenic fault responsible for the 2017 Milin earthquake. This earthquake had a moment magnitude of Mw 6.5 and a maximum slip of approximately 1.08 m and occurred at a depth of approximately 10 km. The numerical simulation of strong ground motions indicates that at the epicenter of the Milin earthquake, the maximum peak ground velocity reached approximately 0.3 m/s. The postseismic effect accelerated the evolution rate of slopes in the Galablaige peak area, thereby further increasing the risk of geological hazards such as landslides and avalanches. The surface strain rate results indicate that the Xixingla fault is in a high-strain zone. The Coulomb stress analysis reveals further stress accumulation along the Jiali fault and Xixingla fault after the 2017 Milin earthquake.

A comprehensive analysis revealed that the mechanism of the 2017 Milin earthquake involved the subduction of the Indian Plate beneath the Eurasian Plate. Consequently, this process led to stress loading in the seismogenic region of the Milin earthquake, which was located within the Eastern Himalayan Syntaxis. Under sustained tectonic stress conditions, the Xixingla fault experienced unstable rupture. The 2017 Milin earthquake reflected tectonic deformation along the southeastern margin of the Tibetan Plateau. However, postseismic effects may accelerate strain accumulation on other active faults in the region, thereby increasing regional seismic risk. Given the future seismic risk in this area, it is imperative to conduct further investigations into the region's seismic activity characteristics.

## Data availability

The teleseismic data used in this study came from the IRIS Data Management Center (http://ds.iris.edu/wilber3/find_event). The Sentinel-1 data used in this study were downloaded from the European Space Agency (ESA) through the ASF Data Hub website https://vertex.daac.asf.alaska.edu/. The SM–VCE software can be accessed through the website https://zenodo.org/records/6,346,205. The Coulomb 3.3 software is also available at https://pubs.usgs.gov/of/2011/1060/. The focal mechanisms were sourced from the Global Centroid Moment Tensor (GCMT) at https://www.globalcmt.org, the United States Geological Survey (USGS) at https://earthquake.usgs.gov/earthquakes/, and the China Earthquake Networks Center (CENC) at http://www.ceic.ac.cn. Seismic events from the period 1900–2023 have been compiled from the USGS (https://earthquake.usgs.gov/), CENC (http://www.ceic.ac.cn), and Xie et al. [24]. The aftershocks form Peng et al. [22]. Coesismic near-field GPS data were taken from Jian et al. [18], while regional surface strain rates were computed using GPS data harnessed from Wang and Shen [12] and Zhang et al. [13]. The Strain_2D software is accessible via https://github.com/kmaterna/Strain_2D. Some figures in this study were generated using the public domain Generic Mapping Tools software [50].

## CRediT authorship contribution statement

**Junyi Wang:** Conceptualization, Data curation, Formal analysis, Methodology, Resources, Software, Validation, Visualization, Writing – original draft. **Shishu Zhang:** Investigation, Writing – review & editing. **Youjia Zhao:** Data curation, Methodology, Software, Visualization. **Fulong Cai:** Data curation, Investigation. **Chao Wang:** Validation, Writing – original draft. **Jiankun He:** Methodology, Writing – original draft. **Lin Ding:** Conceptualization, Data curation, Formal analysis, Funding acquisition, Project administration, Supervision, Validation, Writing – review & editing.

## Appendix Supplementary materials

Image, application 1
