# Supplementary material for: Coseismic deformation analysis of the 2017 Milin Ms 6.9 earthquake in the Namche Barwa Syntaxis: Implications for regional tectonics
Source: Fundam Res. 2024 Sep 17;5(6):2707–18. doi: 10.1016/j.fmre.2024.09.003 (PMC12744612; doi:10.1016/j.fmre.2024.09.003)
Supplement: Supplementary file 1 [file mmc1.docx]

# Supplementary Information for

# Coseismic deformation analysis of the 2017 Milin earthquake in the Namche Barwa Syntaxis: Implications for regional tectonics

Junyi Wang^a,b,1^, Shishu Zhang^c^, Youjia Zhao^a,b^, Fulong Cai^a,b^, Chao Wang^a^, Jiankun He^a,b^, Lin Ding^a,b,1,^*

*^a^ State Key Laboratory of Tibetan Plateau Earth System, Environment and Resources, Institute of Tibetan Plateau Research, Chinese Academy of Sciences, Beijing 100101, China.*

*^b^ University of Chinese Academy of Sciences, Beijing 100049, China.*

*^c^POWERCHINA Chengdu Engineering Corporation Limited, Chengdu 610072, China.*

** Corresponding author:dinglin@itpcas.ac.cn (L. Ding).*

## ^1^ *These authors contributed equally to this work*

**This file includes:**

Figures S1-S5

Table S1


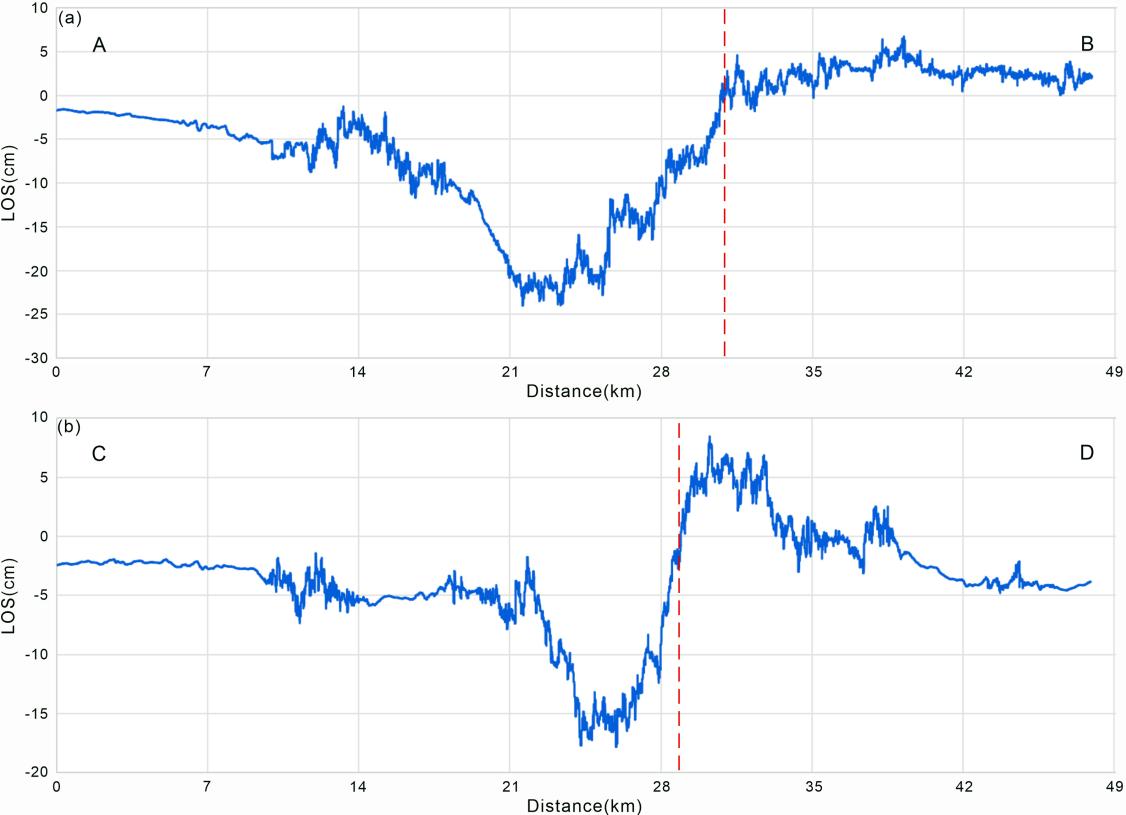


**Fig. S1.** (a) Ascending coseismic deformation profile. (b) Descending coseismic deformation profile.


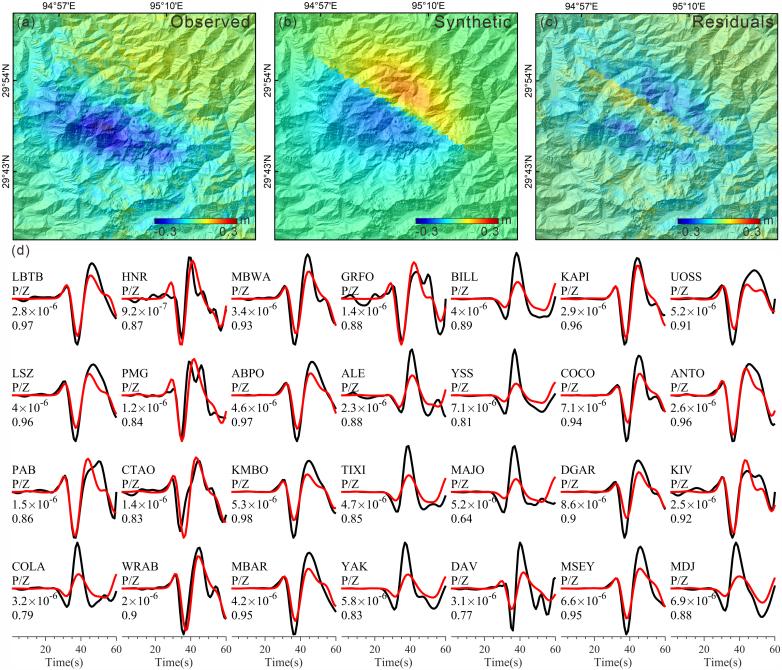


**Fig. S2.** (a) Observed and simulated InSAR data and residuals. (b) Observed (black lines) and synthesized (red lines) teleseismic waveforms.

**
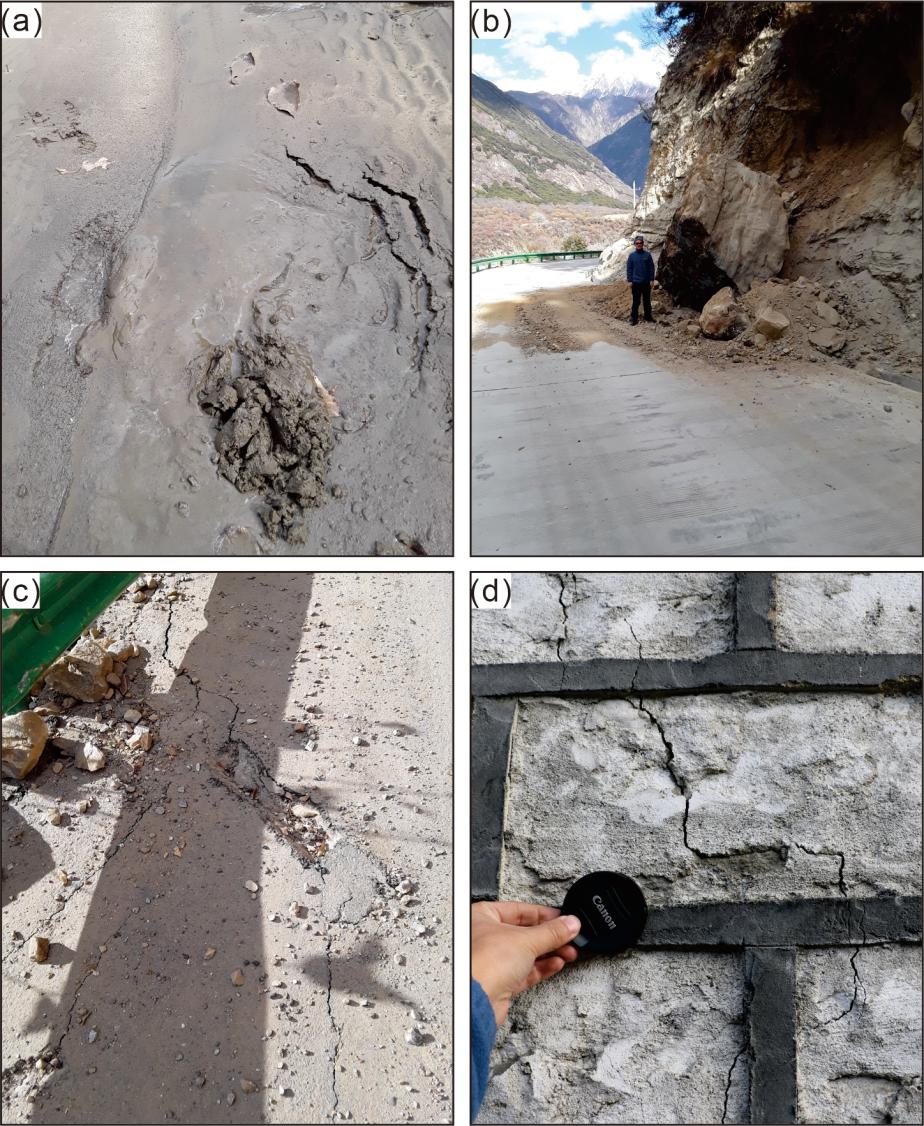
**

**Fig. S3.** (a) Sand liquefaction. (b) Rock collapses. (c) Surface fissures. (d) Cracks in walls.

**
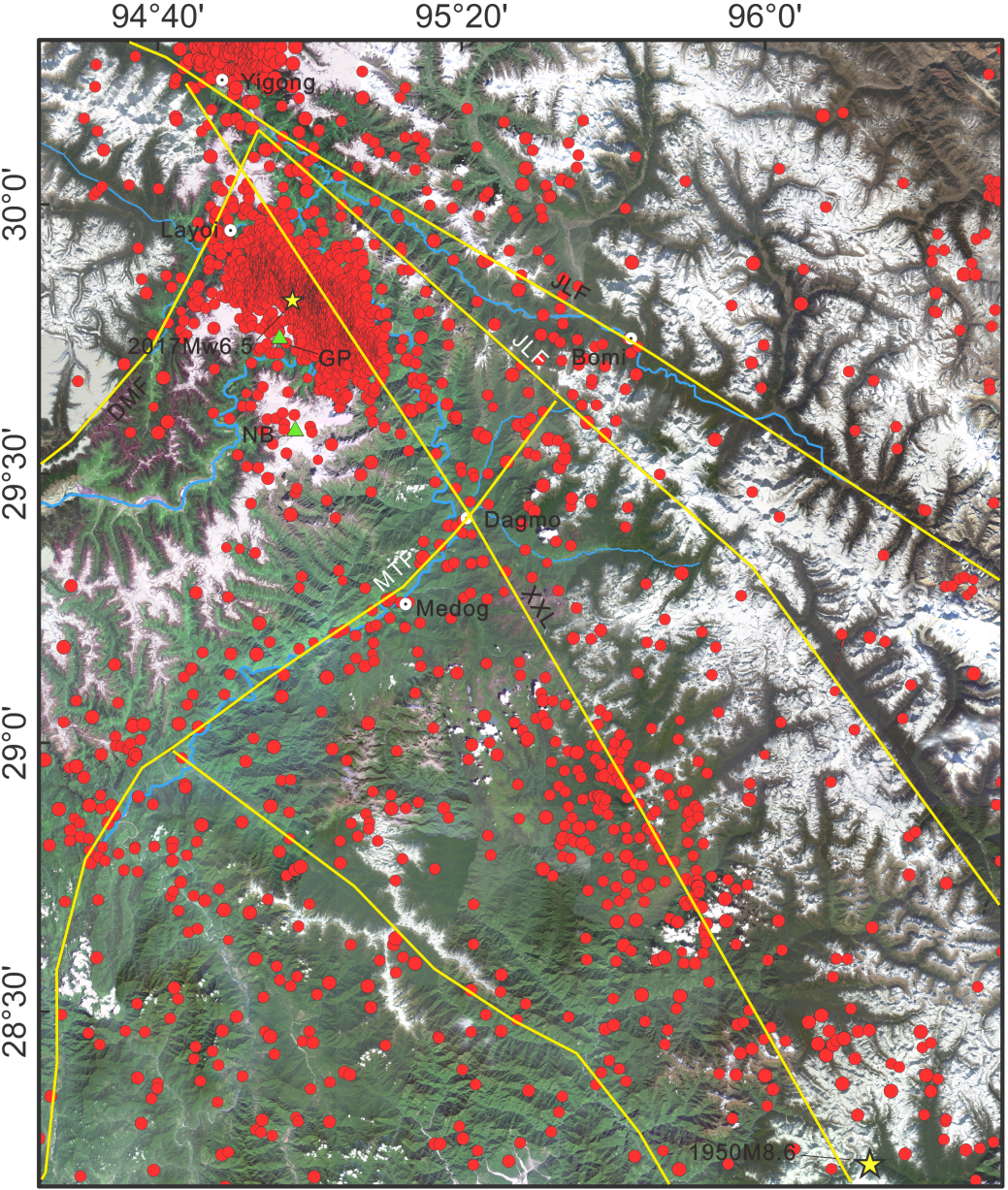
**

**Fig. S4.** Seismic distribution characteristics of the Xixingla fault. The red dots represent historical earthquakes in the region from 1900 to 2023.


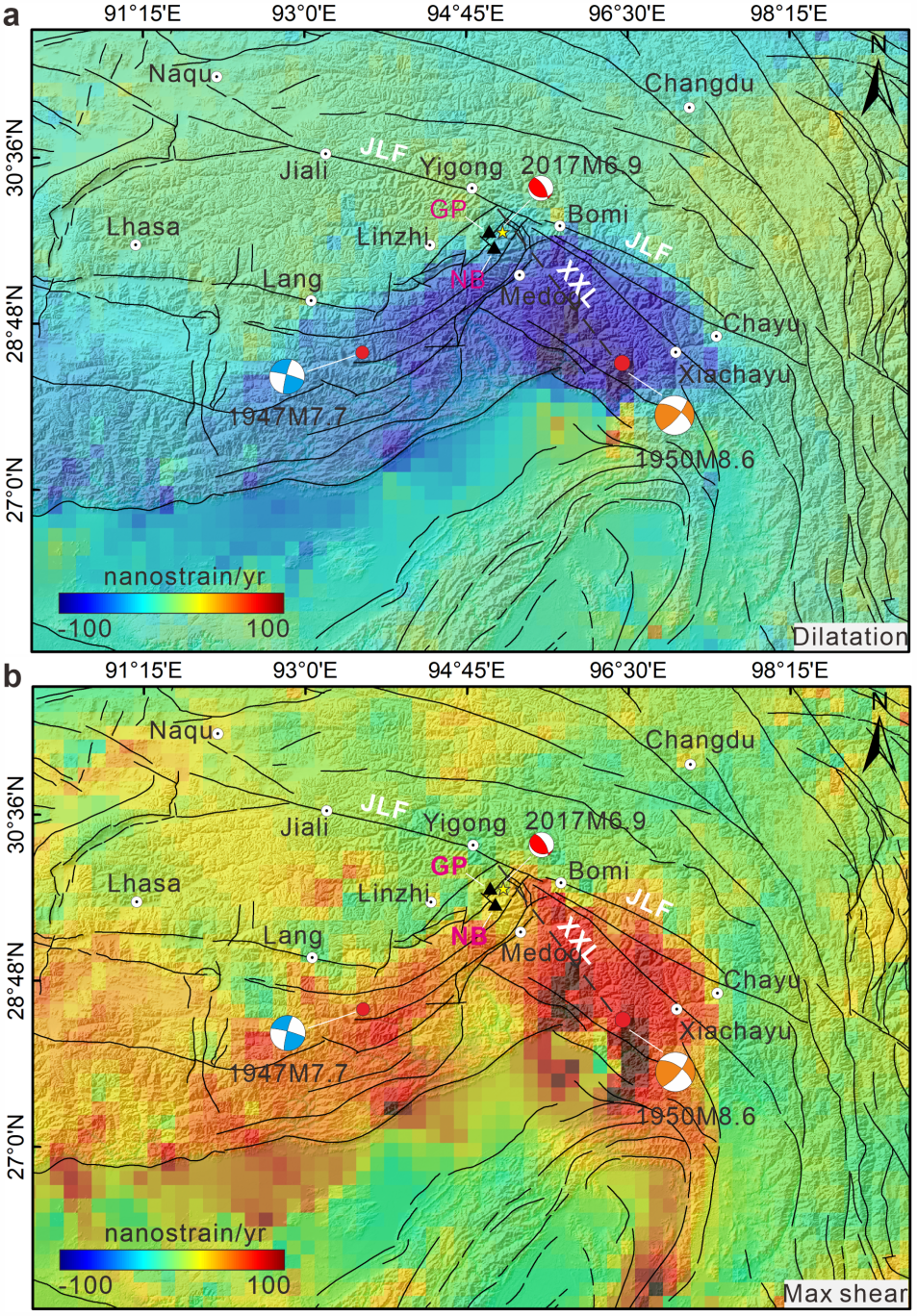


**Fig. S5**. (a) Areal dilatational rate field of the Eastern Himalayan syntaxis and its vicinity. The positive and negative color bands indicate volume expansion and contraction, respectively, in the corresponding regions. (b) Maximum shear rate field of the Eastern Himalayan syntaxis and its vicinity.

**Table S1. SAR images used in the InSAR analysis.**

| **Flight Direction** | **Path** | **Master** | **Slave** | **Time Baseline/d** | **Spatial Baseline/m** | **Incidence Angle/°** |
| --- | --- | --- | --- | --- | --- | --- |
| Ascending | 70 | 2017/11/11 | 2017/11/23 | 12 | 33.8 | 37.44 |
| Descending | 4 | 2017/11/6 | 2017/11/18 | 12 | 9.6 | 40.62 |
